# Treatment-related toxicities of apatinib in solid tumors: a meta-analysis

**DOI:** 10.18632/oncotarget.24215

**Published:** 2018-01-13

**Authors:** Ling Peng, Xianghua Ye, Yun Hong, Junyan Zhang, Yongquan Dong, Qiong Zhao

**Affiliations:** ^1^ Department of Thoracic Oncology, The First Affiliated Hospital, School of Medicine, Zhejiang University, Hangzhou, Zhejiang Province, China; ^2^ Department of Radiotherapy, The First Affiliated Hospital, School of Medicine, Zhejiang University, Hangzhou, Zhejiang Province, China; ^3^ Department of Pharmacy, The First Affiliated Hospital, School of Medicine, Zhejiang University, Hangzhou, Zhejiang Province, China; ^4^ Bothwin Clinical Study Consultant, Bellevue, WA, USA; ^5^ Department of Respiratory Disease, Yinzhou No.2 Hospital, Ningbo, Zhejiang Province, China

**Keywords:** apatinib, hypertension, proteinuria, hand-foot-syndrome, meta-analysis

## Abstract

**Background:**

Apatinib is a novel small molecular drug targeting vascular endothelial growth factor receptor-2 (VEGFR-2), which is being studied in multiple tumor types. We performed a meta-analysis to quantify the overall incidence and risk of hypertension, proteinuria, and hand-foot-syndrome (HFS) in cancer patients receiving apatinib.

**Results:**

Altogether, 820 cancer patients from 7 prospective trials were included for the meta-analysis. The incidences of all-grade and high-grade hypertension were 45.4% and 9.7%. The incidences of all-grade and high-grade proteinuria were 45.1% and 3.7%. The incidences of all-grade and high-grade HFS were 35.9% and 8.6%. The RRs of all-grade hypertension, proteinuria and HFS of apatinib compared to placebo were increased (hypertension, RR = 6.53; proteinuria, RR = 2.62, and HFS, RR = 11.45). The RRs of developing high-grade hypertension and HFS were substantially higher than that of placebo (hypertension, RR = 7.73; HFS, RR = 7.23), but not for proteinuria (RR = 2.56, 95% CI: 0.57–11.52).

**Materials and Methods:**

Prospective phase II and III clinical trials of cancer patients receiving apatinib were searched and included. Summary incidences, relative risk (RR), and 95% confidence intervals (CI) were calculated by using either fixed or random effects models according to the heterogeneity of the studies.

**Conclusions:**

Apatinib is generally well tolerated, and associated with increased risks of all-grade hypertension, proteinuria and HFS, and high-grade hypertension and HFS, but not high-grade proteinuria.

## INTRODUCTION

Apatinib (YN968D1) is a potent small molecule inhibitor of vascular endothelial growth factor receptor-2 (VEGFR-2, Flk-1/KDR) and RET (rearranged during transfection) [1]. Although apatinib shares target receptors with other antiangiogenic drugs, it is highly selective. It is approved as third-line treatment for gastric cancer by Chinese Food and Drug Administration in 2014, based on a multi-center, randomized, phase III trial comparing apatinib 850 mg once daily (QD) versus placebo [2]. The results of the study found that overall survival (OS) was increased for patients receiving apatinib compared with placebo (6.5 vs. 4.7 months, *P* = 0.0149). Currently, apatinib is undergoing clinical trials as single agent and in combination with chemotherapy or immunotherapy for treatment of other types of cancer, such as lung cancer, colorectal cancer, esophageal cancer.

Apatinib is almost free of the classical toxicities of cytotoxic chemotherapy, but other side effects such as hypertension, proteinuria and hand-foot-syndrome, can lead to decreased quality of life (QoL) or interruptions of treatment. The overall incidences and RRs of the most common adverse events of apatinib have not been systematically reviewed. It allows for clinical consideration of specific therapies based on efficacy and toxicity profiles. Therefore, we conducted this meta-analysis to quantify the incidence and relative risk of the three most toxicities (hypertension, proteinuria and hand-foot-syndrome) among cancer patients receiving apatinib.

## RESULTS

### Study selection and characteristics

**Figure 1:**
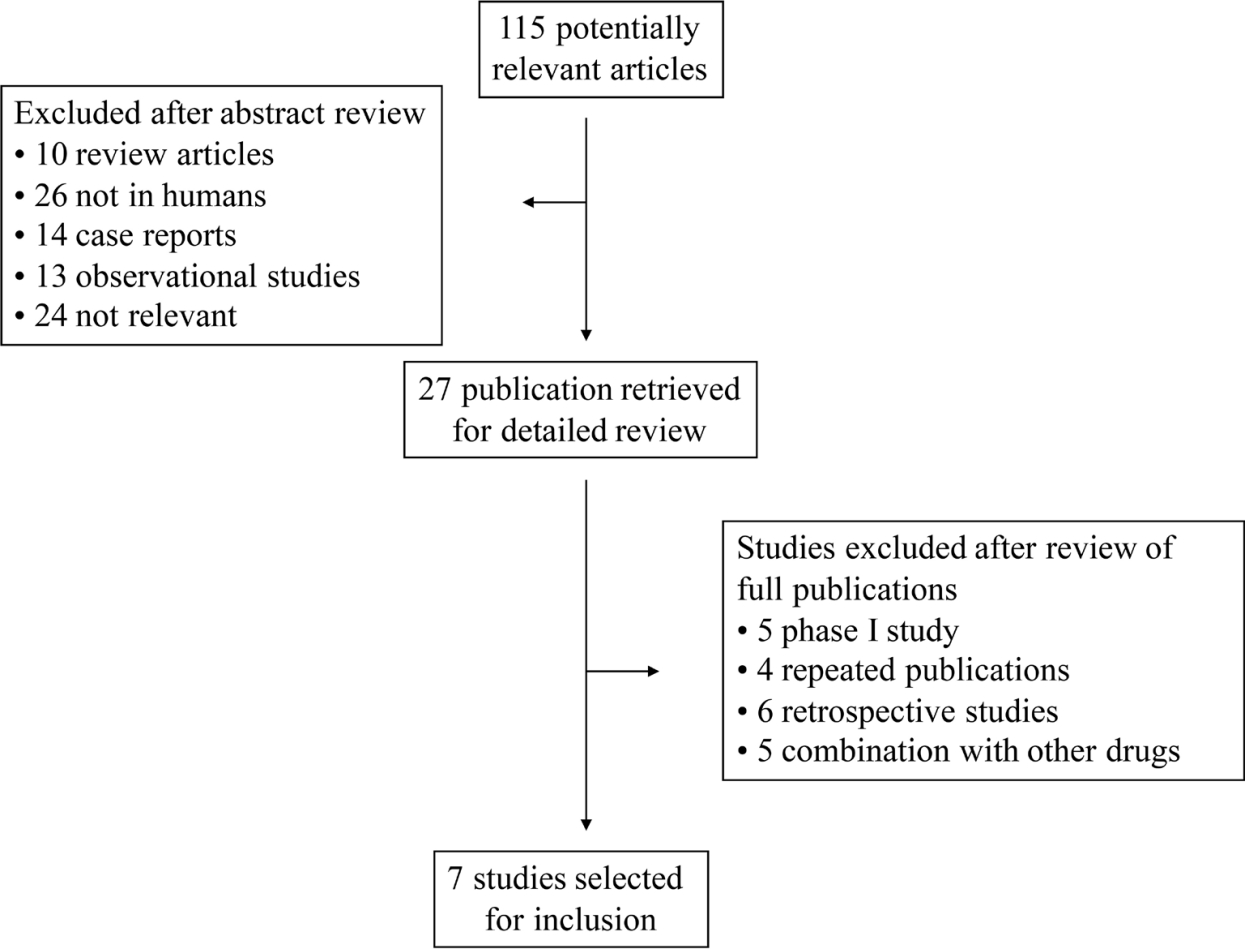
Selection process for the trials included in the meta-analysis

Our comprehensive search of the literature revealed 81 potentially relevant records. After screening of the study titles and abstracts 56 studies were excluded, as those were not prospective trials. After text review 20 more studies were excluded for not meeting the inclusion criteria. Five studies met the inclusion criteria and data were extracted. Among the 34 abstracts published in ASCO meetings, 2 abstracts were identified. Altogether, 7 primary studies comprising 820 patients were included for analysis (Figure 1). The baseline information of the 7 primary studies were shown in Table 1, including 1 randomized controlled trials (RCTs) and 6 phase II clinical trials. Underlying malignancies include gastric cancer (2 trials) [2, 3], lung cancer (2 trials) [4, 5], breast cancer (2 trials) [6, 7] and hepatocellular carcinoma (1 trial) [8]. The sample size ranged from 33 to 267 patients (median, 121 patients). The studies were published between 2012 and 2017, and all the included studies were performed in China. For calculation of the RRs, 3 RCTs were pooled [2–4]. The meta-analysis adheres to the guidelines of the Preferred Reporting Items for Systematic review and Meta-Analyses (PRISMA) statement [9].

**Table 1:** Main characteristics and results of the eligible studies

Year	Study	Phase	Research	Cancer type	Source	Treatment Arm	Hypertension all-grade		Hypertension high-grade		Proteinuria all-grade		Proteinuria high-grade		HFS all-grade		HFS high-grade		Patients
2017	Wang [5]	2	Single arm	NSCLC	ASCO	Apatinib 250 mg QD	10	33.3%	0	0.0%	8	24.2%	0	0.0%	5	15.2%	0	0.0%	33
2016	Li [2]	3	RCT	Gastric	Pubmed	Apatinib 850 mg QD	62	35.2%	8	4.5%	84	47.7%	4	2.3%	49	27.8%	15	8.5%	176
						Placebo	5	5.5%	0	0.0%	15	16.5%	0	0.0%	2	2.2%	0	0.0%	91
2014	Qin [8]	2	Parallel arm	HCC	ASCO	Apatinib 850 mg QD	35	50.0%	2	2.9%	32	45.7%	1	1.4%	29	41.4%	4	5.7%	70
						Apatinib 750 mg QD	25	49.0%	7	13.7%	22	43.1%	2	3.9%	15	29.4%	4	7.8%	51
2014	Hu [7]	2	Parallel arm	TNBC	Pubmed	Apatinib 750 mg QD	15	60.0%	9	36.0%	16	64.0%	1	4.0%	14	56.0%	0	0.0%	25
						Apatinib 500 mg QD	38	64.4%	7	11.9%	31	52.6%	8	13.6%	14	56.0%	6	24.0%	59
2014	Hu [6]	2	Single arm	Non TNBC	Pubmed	Apatinib 500 mg QD	16	42.1%	8	21.1%	20	52.6%	2	5.3%	20	52.6%	4	10.5%	38
2013	Li [3]	2	RCT	Gastric	Pubmed	Apatinib 850 mg QD	19	40.4%	4	8.5%	13	26.7%	1	2.1%	12	25.5%	2	4.3%	47
						Apatinib 425 mg BID	18	39.1%	5	10.9%	16	34.8%	2	4.3%	21	45.7%	6	13.0%	46
						Placebo	2	4.2%	0	0.0%	6	12.5%	0	0.0%	2	4.2%	1	2.1%	48
2012	Zhang [4]	2	RCT	NSCLC	ASCO	Apatinib 750 mg QD	42	46.2%	4	4.4%	46	50.6%	2	2.2%	30	33.0%	4	4.4%	91
						Placebo	4	8.9%	0	0.0%	10	22.2%	1	2.2%	1	2.2%	0	0.0%	45

Summary table of studies included in the meta-analysis. Abbreviations: CI, confidence interval; NR, not reported.

### Incidence of hypertension, proteinuria and HFS

**Figure 2:**
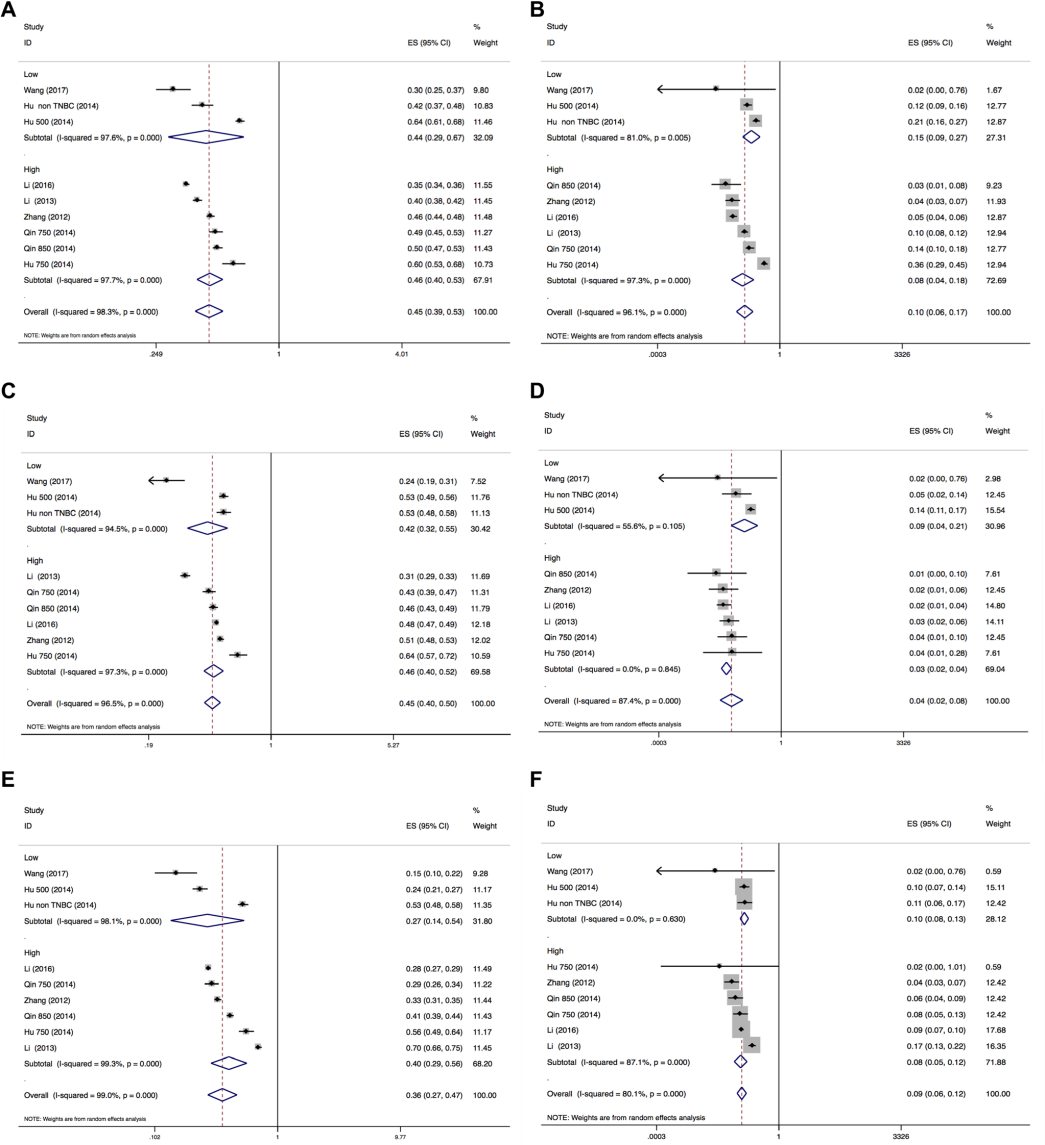
Forest plot for meta-analysis of incidence of all-grade and high-grade hypertension, proteinuria and HFS (dose subgroup) Each study was shown by the name of the lead author and year of publication. The summary incidence was also shown in the figure. Plots are arranged as follows: (**A**) Incidence of all-grade hypertension; (**B**) Incidence of high-grade hypertension. (**C**) Incidence of all-grade proteinuria; (**D**) Incidence of high-grade proteinuria. (**E**) Incidence of all-grade HFS; (**F**) Incidence of high-grade HFS.

**Figure 3:**
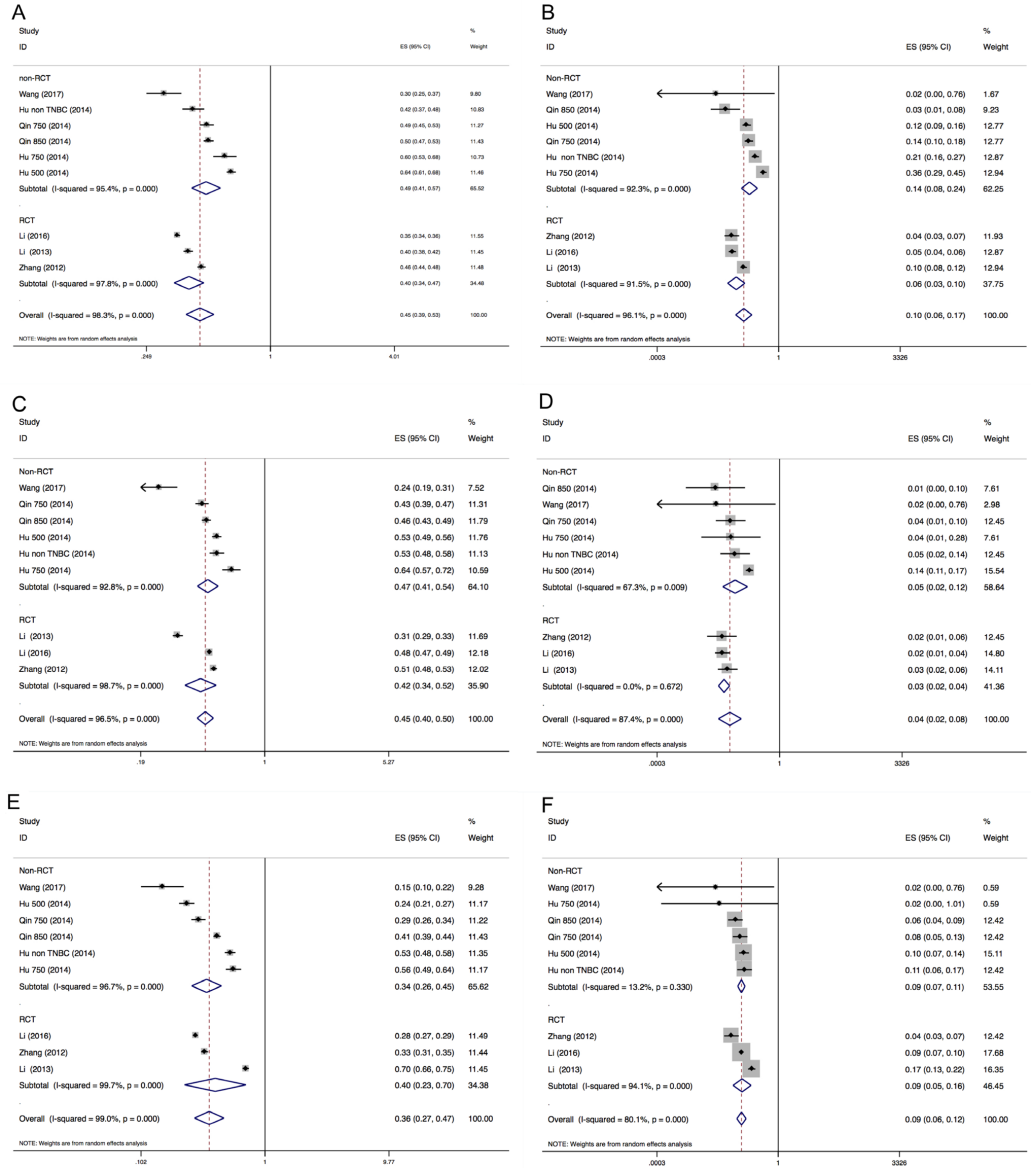
Forest plot for meta-analysis of incidence of all-grade and high-grade hypertension, proteinuria and HFS (research type subgroup) Each study was shown by the name of the lead author and year of publication. The summary incidence was also shown in the figure. Plots are arranged as follows: (**A**) Incidence of all-grade hypertension; (**B**) Incidence of high-grade hypertension. (**C**) Incidence of all-grade proteinuria; (**D**) Incidence of high-grade proteinuria. (**E**) Incidence of all-grade HFS; (**F**) Incidence of high-grade HFS.

The results of the incidences of hypertension, proteinuria and HFS were shown in Figure 2 and Figure 3. A total of 820 patients from 7 trials were included for analysis of incidence. The summary incidence of all-grade and all-grade hypertension was 45.4% (95% CI: 38.8–53.0%) and 9.7% (95% CI: 5.7–16.7%) using a random effects model, respectively. Inter-study heterogeneity *I*^2^ statistics were 98.3% for all grade hypertension (*P* < 0.001), 96.1% for high-grade hypertension (*P* < 0.001), 96.5% for all-grade proteinuria (*P* < 0.001), 87.4% for high-grade proteinuria (*P* < 0.001), 99.0% for all-grade HFS (*P* < 0.001), and 80.1% for high-grade HFS (*P* < 0.001), respectively. No statistically significant subgroup difference was identified in terms of dose (≥ 750 mg QD vs < 750 mg QD, Figure 2) or clinical trial type (RCT vs non-randomized trial, Figure 3).

### Relative risk of hypertension, proteinuria and HFS

**Figure 4:**
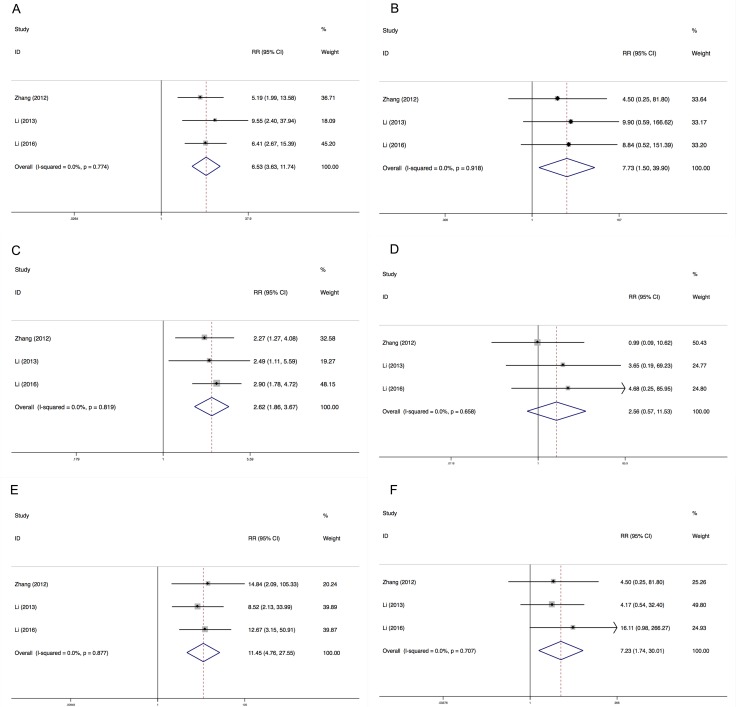
Relative risks of all-grade and high-grade hypertension, proteinuria and HFS Each study was shown by the name of the lead author and year of publication. Plots are arranged as follows: (**A**) Relative risk of all-grade hypertension; (**B**) Relative risk of high-grade hypertension. (**C**) Relative risk of all-grade proteinuria; (**D**) Relative risk of high-grade proteinuria. (**E**) Relative risk of all-grade HFS; (**F**) Relative risk of high-grade HFS.

We determined the relative risks (RRs) of treatment-related toxicities compared with control arm. In the included studies, all control arms were placebo. The pooled RR showed that apatinib increased the risk of developing all-grade and high-grade hypertension with a RR of 6.53 (95% CI: 3.63–11.73, *I*^2^ = 0.0%, *P* = 0.774), and 7.73 (95% CI: 1.50–39.90, *I*^2^ = 0.0%, *P* = 0.918), using a fixed effects model, respectively (Figure 4). The RR of HFS was also increased for all-grade (RR = 11.45, 95% CI: 4.76–22.55, *I*^2^ = 0.0%, *P* = 0.877) and high-grade (RR = 7.23, 95% CI: 1.74–30.01, *I*^2^ = 0.0%, *P* = 0.707). However, apatinib was associated with increased risk of all-grade proteinuria (RR = 11.45, 95% CI: 4.76–22.55, *I*^2^ = 0.0%, *P* = 0.819) but not high-grade proteinuria (RR = 2.56, 95% CI: 0.57–11.52, *I*^2^ = 0.0%, *P* = 0.658).

### Sensitivity analysis

**Figure 5:**
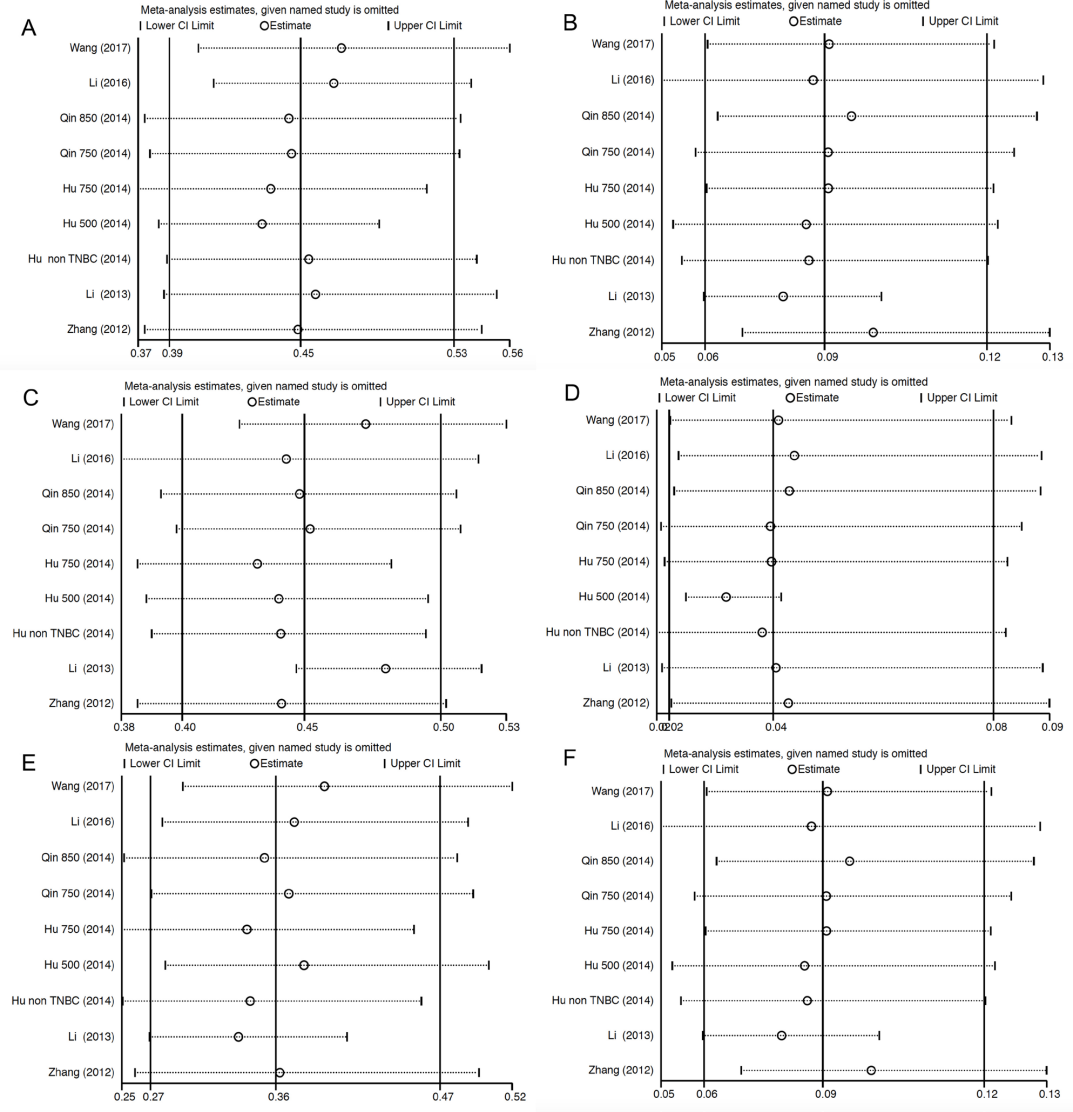
Sensitivity analysis for studies included in the meta-analysis for analysis of relative risk Plots are arranged as follows: (**A**) Sensitivity analysis of incidence of all-grade hypertension; (**B**) Sensitivity analysis of incidence of high-grade hypertension. (**C**) Sensitivity analysis of i of all-grade proteinuria; (**D**) Sensitivity analysis of incidence of high-grade proteinuria. (**E**) Sensitivity analysis of incidence of all-grade HFS; (**F**) Sensitivity analysis of incidence of high-grade HFS.

Sensitivity analysis was performed to test the robustness and stability of our results. The significance estimate of pooled results was not significantly influenced by omitting any single study (Figure 5).

### Publication bias

Seven studies reporting all-grade and high-grade hypertension yielded an Egger’s test score of *P* = 0.405 and *P* = 0.383, respectively. No evidence of publication bias was detected for the incidence of all-grade and high-grade proteinuria of this study (*P* = 0.570 and *P* = 0.054, respectively). The Egger’s test scores for all-grade and high-grade proteinuria were *P* = 0.826 and *P* = 0.538, respectively.

## DISCUSSION

VEGF signaling pathway plays an important role in the angiogenic process of cancer. VEGFR-2 is auto-phosphorylated when stimulated by VEGF, which is the most pro-angiogenic effect [10]. VEGFR-2 inhibitors have been developed, including receptor-specific antibodies and tyrosine kinase inhibitors. Among them, apatinib is a selectively inhibitor which targets VEGFR-2 and inhibits c-Kit and c-Src tyrosine kinases. It is approved by Chinese FDA in 2014 for the use in third-line treatment with gastric cancer patients who progressed after second-line chemotherapy. Its application in other cancer types is also under clinical investigation.

Apatinib is generally well tolerated. However, drug-related toxicities and adverse effects also developed, which are manageable. The major non-hematological adverse effects reported of this drug are hypertension, proteinuria, and HFS, but incidences vary substantially among clinical trials. Management include dose reduction, interruption, or termination of the drug. The time to onset of in the hypertension, proteinuria, and HFS apatinib groups was within 2 to 3 weeks. Due to the small molecule nature of apatinib, the time to resolution of above mentioned side effects was faster than those of bevacizumab. Of note, supportive treatment, which could have contributed to resolution of high-grade AEs could be a confounding factor for time to resolution of AEs.

The purpose of this study is to quantify the incidences and relative risks of three common toxicities of hypertension, proteinuria and HFS in cancer patients receiving apatinib. This meta-analysis combined 7 studies including 1 phase III trials and 6 phase II trials. The results demonstrated that apatinib is associated with increased risks of developing high-grade hypertension and HFS, but not proteinuria. The overall incidence of all-grade and high-grade hypertension, proteinuria, and HFS were summarized in the present meta-analysis. The relative risks of all-grade hypertension, proteinuria and HFS were significantly increased for 6-, 2-, and 11-fold, respectively. As for high-grade toxicities, compared with placebo, apatinib had 7-fold risk of developing hypertension and HFS, but not proteinuria. The confidence interval generated from the forest plot (Figure 2 and Figure 3) indicated the relatively small sample size of this meta-analysis. Therefore, larger randomised clinical trials are warranted to reach firm conclusion. Since single agent is sufficiently effective, combination therapy are the solution to improve the outcome. At present, several clinical trials are undertaking to combine apatinib with chemotherapy, immunotherapy and other anti-neoplastic drugs. A phase 4 clinical trial is undergoing to assess the safety and efficacy of apatinib in patients with chemo-refractory gastric cancer in clinical practice (NCT02426034).

These toxicities were also reported in other multikinase inhibitors, such as sorafenib [11], sunitinib [12], pazopanib [13, 14], axitinib [15, 16], and regorafenib [17, 18], etc. Our finding is consistent with other anti-angiogenesis drugs. VEGF plays an important part in regulating glomerular vascular permeability. Inhibition of VEGF-dependent interactions between podocytes and glomerular endothelial cells disrupts the filtration barrier, which results in proteinuria [19]. The development of hypertension is thought be an on-target effect of the VEGF inhibitor; therefore, it could be considered as a potential predictive factor of oncologic response [20, 21]. The development of HFS appears to correlate with dose escalations of the multikinase drug, which suggests that this may be due to a direct mechanism-based effect [22]. Early onset adverse effects of apatinib is reported to be predictive of its efficacy [23], which highlighted the importance of ensuring efficacy and maintaining QoL at the same time.

Our meta-analysis demonstrates that apatinib-associated major toxicities are mostly grade 1 and 2. The manufacturer instruction of apatinib recommends monitoring for adverse events. For patients with high-grade hypertension, proteinuria and HFS, apatinib should be discontinued. If symptoms relieved within 2 weeks, then the original dose can be administered when adverse event dropped to below grade 2. If the adverse events continued for more than 2 weeks, dose reduction was recommended.

Our meta-analysis is not without limitations. First, the included studies were conducted with various types of cancer patients in different centers, which may have potential bias in recording adverse events. Secondly, the dose of apatinib used in clinical trial varied from trial to trial, and the dose reduction of apatinib were allowed in clinical trials, which may interfere with the results. Thirdly, there was heterogeneity among primary studies regarding small sample size and the limited primary studies.

Despite the above limitations, our meta-analysis is the first one to systematically study the incidence and relative risk of hypertension, proteinuria and HFS associated with apatinib in cancer patients. These toxicities were modest and manageable, and the results would provide important information for clinicians.

## MATERIALS AND METHODS

### Search strategy and study selection

Databases of PubMed, Embase, and Cochrane were searched for relevant studies (up to June 2017). Abstracts presented at ASCO annual meetings were also searched. Search terms include: (“apatinib”, OR “YN968D1”) And (“cancer”, OR “carcinoma”, OR “sarcoma”), And (“clinical trial”). Apatinib was approved for previously treated gastric cancer patients with at a recommended dose of 850 mg QD by Chinese FDA. Clinical trials using apatinib at different doses were included in the study to assess the incidence and relative risk with these treatments.

Relevant studies that met the following criteria were included: (1) prospective phase II and III clinical trials in cancer patients; (2) participants assigned to single agent apatinib; (3) the search was restricted to clinical trial published in English; (4) events or event rate and sample size available for hypertension, proteinuria, and HFS, and (5) if multiple publications of the same trial were retrieved, only the most informative one was included. Trials with relatively small number of patients (less than 20) were excluded. Abstracts were read by two authors (LP and XY) independently. Articles that could not be determined based on title and abstract were reviewed of full-text.

### Study selection

Data abstraction was conducted by two investigators (LP and XY) independently. Toxicity profile including hypertension, proteinuria and hand-foot-syndrome was extracted from the primary study. These clinical end points were recorded according to versions 4.0 of the Common Terminology Criteria for Adverse Events of National Cancer Institute (NCI-CTCAE). We included all incidences of hypertension, proteinuria and hand-foot-syndrome of grade 1 or above in our analysis.

### Data analysis

Information was retrieved from the primary studies, including the following items: publication year, first author, cancer type, clinical trial phase, sample size, treatment arm, and control arm. Toxicity data of all-grade and high-grade (grade 3/4) of hypertension, proteinuria, and HFS and the number of patients receiving apatinib were extracted. We derived the proportion and calculated the 95% CI of patients with hypertension, proteinuria, and HFS from each study. To calculate the summary incidence, we used an inverse variance statistical method. For controlled studies, we calculated and compared the RRs. For one study that reported zero event in control arm, half-integer correction was used [24].

The *χ*^2^-based *Q* statistic was used to calculate the heterogeneity between selected studies [25]. Heterogeneity was considered to be statistically significant if *P* < 0.10 or *I*^2^ > 50%. If heterogeneity existed, data were analyzed using a random-effects model, otherwise, a fixed-effects model was used.

A two-sided *P* value of < 0.05 was considered statistically significant. The presence of publication bias was evaluated by using the Begg’s and Egger’s tests [26, 27]. To assess the stability of results, we performed sensitivity analysis by sequential omission of individual study. All of the calculations were performed by STATA version 14.0 (Stata Corporation, College Station, TX).
